# Prevalence and risk factors determinants of the non-use of insecticide-treated nets in an endemic area for malaria: analysis of data from Cameroon

**DOI:** 10.1186/s12936-023-04510-9

**Published:** 2023-07-05

**Authors:** Magloire T. C. Kuetche, Raymond N. Tabue, C. D. Fokoua-Maxime, Armel M. Evouna, Serge Billong, Olivier Kakesa

**Affiliations:** 1 National Malaria Control Programme, Yaoundé, Cameroon; 2 School of Public Health, New York State University at Albany, Albany, NY USA; 3 Cameroon Field Epidemiology Training Program, Yaoundé, Cameroon; 4 National Committee Against HIV-AIDS, Yaoundé, Cameroon; 5 President’s Malaria Initiative (PMI)-Measure Malaria Project, Yaoundé, Cameroon

**Keywords:** ITNs, Non-use, Prevalence, Determinants, Cameroon

## Abstract

**Background:**

Malaria is the main cause of morbidity and mortality in Cameroon. Insecticide-treated nets (ITNs) significantly reduce malaria transmission, but their use is not common in the population. This study aimed to estimate the nationwide prevalence of the non-use of ITNs and identify its major determinants.

**Methods:**

A cross-sectional study was conducted on interview data collected in households selected across all the regions of Cameroon through a non-probabilistic, random, 2-stage stratified sampling process. Descriptive statistics were used to describe the distribution of baseline characteristics across the households, and statistical tests assessed if the distribution of these characteristics differed significantly based on the non-use of ITNs, with 0.05 serving as a threshold of the p-value for statistical significance. The prevalence of the non-use of ITNs was estimated, and logistic regression models were used to tally the odds ratios of the associations between various factors and the non-use of ITNs, along with their 95% confidence intervals. The sensitivity, specificity, and area under the receiver operating characteristic (ROC) curve (AUC) were determined, and the Hosmer Lemeshow test was used to measure the goodness of fit of each statistical model.

**Results:**

Of the 7593 households interviewed, 77% had at least one ITN and 59% of the population used ITNs. Only 72% of the population with at least one ITN used it. The logistic model of the multivariate analysis was significant at a 5% threshold. The AUC was 0.7087 and the error rate was 18.01%. The sensitivity and specificity of the model were 97.56% and 13.70%, respectively. The factors that were associated with ITN use were the presence of sufficient nets in the household (p < 0.0001), the region of residence (p < 0.0001), the level of education of the respondent (p < 0.0001), and the standard of living (p = 0.0286). Sex, age, colour preferences, as well as the shape and size of the nets were not associated with ITN use.

**Conclusions:**

The use of ITNs in Cameroon was low and varied according to specific factors. These identified factors could be used as the foundations of effective sensitization campaigns on the importance of ITNs.

**Supplementary Information:**

The online version contains supplementary material available at 10.1186/s12936-023-04510-9.

## Background

Malaria is a major public health problem in Cameroon. In fact, malaria was incriminated in 24% of hospital visits, 47% of hospitalizations, and 18% of all deaths reported in Cameroon in 2017 [1]. In the same year, the total number of malaria cases was estimated at 7,307,515 [1]. Owing to these high statistics, the World Health Organization (WHO) ranked Cameroon as the 11th country most affected by malaria in the world [2]. Besides the high mortality due to malaria, the disease also has a significant economic impact by imposing substantial costs to both individuals and their families through the purchase of drugs for treating malaria at home; lost days of work; absence from school; expenses for preventive measures [3]. Given these severely detrimental effects of malaria, the government of Cameroon has made malaria control one of its top priorities. Indeed, the country is now engaged in its 5th National Strategic Plan for the Control of Malaria, which primarily focuses on vector control through the free distribution of insecticide-treated nets (ITNs) and the promotion of their use by the entire population [4].

ITNs are a cost-effective and efficient preventive measure against malaria, and their efficacy has been demonstrated in various circumstances [5, 6]. Indeed, evidence suggests that high levels of ITNs ownership and use by all members of a community substantially reduce the vectorial capacity and size of the malaria parasite reservoir [7], and even protect those who do not use ITNs [8]. Hence, ITNs significantly reduce the risk of simple and severe malaria and thus decrease malaria mortality rates in communities that use them [9–14]. In addition, ITNs are highly effective in vulnerable populations. Actually, communities that adopt the use of ITNs observe a 25% average reduction in child mortality [13]. In addition, studies report that the use of ITNs among pregnant women is associated with a lower prevalence of malaria infection, fewer premature births, and a significant reduction in maternal anemia and placental infection, which all result in lower maternal and infant mortality rates [15–19]. Owing to the aforementioned positive effects, the WHO recommends ITNs as the primary prevention measure against malaria in endemic areas [20].

Cameroon is an endemic area for malaria, and the use of ITNs has long been promoted. While the free access to ITNs was for years reserved for pregnant women, the last decade saw the government of Cameroon and its international partners take the initiative to provide free access to ITNs to all the households of Cameroon, through nationwide distribution campaigns. These campaigns were conducted in 2011 and 2015–2016, during which a total of 8,115,879 and 11,790,598 ITNs were distributed, respectively [21]. The direct effect was a substantial increase in the ITNs possession rates. Nationwide surveys reported that the ITNs possession rates increased from 33% in 2011 to 66% in 2013 and 77% in 2017 [24, 25]. Despite these high possession rates, the proportion of households actually using the ITNs has remained relatively low, at 13% in 2011 [24], 39% in 2013 [25], and 58% in 2017 [24]. These small proportions of ITNs usage are significantly lower than the objective of Cameroon health authorities to get at least 80% of the population to sleep under ITNs [25]. Furthermore, the low usage of ITNs might in part explain why the mortality and morbidity of malaria remain high in Cameroon, depriving the country of populations and resources much needed for its development. Thus, it is important to obtain an updated estimate of the nationwide prevalence of the non-use of ITNs in Cameroon and to identify its major determinants so that they can be controlled – this objective was addressed in the present study.

## Methods

### Study site

Cameroon is a sub-Saharan African country located above the equator between latitude 6°N and longitude 12°E, and it hosts a population which was estimated at 25,876,387 inhabitants in 2019 [26]. The populations live primarily in rural areas, but the massive rural exodus has caused a substantial demographic expansion of urban centers where living conditions are poor because of no a lack of urban planning. In terms of geography, Cameroon is characterized by a diverse natural environment spreading across its different regions [27]. The country displays two main climate types (tropical and equatorial) which are further subdivided into four sub-types; (i) the Sahelian climate in the Far North with a rainfall average between 400 and 900 mm and temperatures averaging 28 °C, (ii) the Sudanese tropical climate in the North with average rainfall between 900 and 1500 mm and temperatures averaging 28 °C, (iii) the equatorial climate in the Southern, Central and Eastern regions characterized by two dry seasons and two rainy seasons with abundant rainfall (between 1500 and 2000 mm) and temperatures averaging 25 °C, and finally (iv) the Cameroonian equatorial climate in the coast and western highlands with abundant rainfall (between 2000 and 10,000 mm) and temperatures averaging 26 °C [28, 29]. Hence, Cameroon constitutes an ideal ecological niche for some *Anopheles* mosquitoes, which are malaria vectors. Indeed, about fifty *Anopheles* species have been identified in Cameroon, and sixteen of them have shown the potential to develop and transmit the human *Plasmodium* [28, 30]. The occurrence, abundance and composition of major vectors tend to vary greatly with the eco-epidemiological settings [31–33]. Although some vectors are described as secondary, they could be primarily responsible for malaria transmission at the local level [34, 35].

### Sampling procedure

A cross-sectional study was conducted on households selected following a two degrees non-probabilistic random stratified sampling procedure. The regions surveyed and the place of residence constituted the two strata. A total number of 12 regions were surveyed. The cities of Yaoundé and Douala (which normally belong respectively to the Center and Littoral regions) have been raised into regions because of their high population densities. Thus, the regions surveyed were namely Adamaoua, East, Far North, North, Northwest, West, South, Southwest, Center, Littoral, Douala and Yaoundé. The categories of the place of residence were urban and rural.

In the first stage, clusters or (EA) were drawn from the twelve surveyed regions from the list of EA established during the third General Population and Housing Census (RGPH) in 2005 [36]. Then a systematic draw was conducted with a probability proportional to size which corresponds to the number of households in the EA; hence, 203 clusters in urban and 158 in rural areas were selected for a total number of 361 clusters.

The counting of households in each of these clusters provided a list of households from which a sample of households was drawn at the second stage with a systematic equal draw probability. Thus, 20 and 25 households were drawn in urban and rural EA, respectively.

### Data collection

The data were collected through a structured interviewer-administered questionnaire. This questionnaire was used to collect information on demographic characteristics (including sex, age, date of birth, educational level) of household members, ownership and usage of nets, evaluation of the ITNs mass campaign distribution, preferences, and continuous use of the mosquito nets. The survey was made in 3 phases according to the implementation of the mass campaign distribution of ITNs, from 19 August to 17 September 2016 for phase 1, from 31 May to 21 June 2017 for phase 2 and from 07 November to 07 December 2017 for phase 3.

### Data analysis

Before data analysis, the factors that could potentially influence the use of Long-lasting insecticidal nets (LLINs) were listed. Among these factors, institutional factors such as availability of health infrastructures and quality of supplies were identified [37], as well as socio-economic factors such as the place of residence [38], the respondent's educational level [39, 40], and the standard of living of the household [41]. The age of the respondent and household size [42, 43] were also thought to influence the possession and use of LLINs. Other factors that could influence LLIN use were their availability in sufficient quantities for universal coverage following the WHO recommendations [44, 45], and the populations’ preferences for certain characteristics of the LLINs.

### Univariate analysis

This consisted of a descriptive analysis of factors that could influence the use of LLINs. A univariate analysis was used to calculate the tendency (mean, median, mode) and dispersion (standard deviation, minimum, maximum) parameters of each factor.

### Multivariate analysis

This firstly consisted of bivariate analyses between the use of LLINs and each of the factors. For each factor, the independence test *χ*^*2*^ between this factor and the LLIN usage variable was calculated. This test allowed to verify the absence/presence of a statistical link between two variables X and Y. The two variables were said to be independent when there was no statistical link between them, and therefore, the knowledge of X did not allow any way of pronouncing on Y. The assumptions of this test were as follows:

$$\left\{\begin{array}{c}{H}_{0}:both\,variables\,X\,and\,Y\,are\,independant\\ {H}_{1}: there\,is\,a\,link\,between\,variables\,X\,and\,Y\end{array}\right.$$

Further, the unadjusted odds ratios and 95% confidence intervals of the association between each of these factors and the use of LLINs were calculated. This allowed to determine the risk factors for the non-use of LLINs and the factors favouring its use within households.

In a second step, multivariate logistic regression was computed to identify the factors related to the use of LLINs while simultaneously accounting for the effect of other determinants. The formulation of the model was as follows:

Let Y be the dependent variable which is the use of LLINs, Y is defined as follows:

$${Y}^{i}=\left\{\begin{array}{c}1\,if\,an\,individual\,i\,has\,slept\,on\,LLIN \\ 0\,if\,an\,indididual\,i\,has\,not\,slept\,on\,LLIN \end{array} \right.$$

$X=\left({X}_{1},\dots , {X}_{k}\right)$ denotes the set of independent variables used in the regression.

On a sample N, ${n}_{1}$ represents the set of individuals sleeping on LLINs ie ${Y}^{i}=1$ and${n}_{2}=N-{n}_{1}$ the set of individuals sleeping on LLINs ie ${Y}^{i}=0$.

P(Y = 1)) (respectively P (Y = 0)) is the prior probability that Y = 1 (respectively Y = 0) P(X│Y = 1) (respectively P (X│Y = 0)) is the conditional distribution of X knowing the value taken by Y.

The posterior probability of obtaining the category 1 of Y (respectively 0) knowing the value taken by X is denoted P (Y = 1│X) (respectively P (Y = 0│X)).

The formulation of the model is given by $\mathrm{ln}\left(\frac{\mathrm{P}\left(\mathrm{X}|\mathrm{Y}=1\right)}{\mathrm{P}\left(\mathrm{X}|\mathrm{Y}=0\right)}\right)={b}_{0}+{b}_{1}{X}_{1}+\dots + {b}_{k}{X}_{k}$

Where $$\frac{\mathrm{P}\left(\mathrm{X}|\mathrm{Y}=1\right)}{\mathrm{P}\left(\mathrm{X}|\mathrm{Y}=0\right)}={e}^{{b}_{0}+{b}_{1}{X}_{1}+\dots + {b}_{k}{X}_{k}}$$

The estimation of logistic regression parameters was done by the maximum likelihood method.

If $$\frac{\mathrm{P}\left(\mathrm{X}|\mathrm{Y}=1\right)}{\mathrm{P}\left(\mathrm{X}|\mathrm{Y}=0\right)}>1\,then\,Y=1\,otherwise\,Y=0$$

The quality of the model was appreciated by several parameters such as the area under the ROC curve, the error rate, the sensitivity, the specificity, and the accuracy. The factors selected were those whose coefficient of regression was significant at 5%. The Odds Ratio of each factor was calculated and analyzed according to 3 criteria that were the meaning, the degree, and the significance of the association.

### Statistics

Excel sheet of Microsoft Office 2010 software was used for data entering and the results generated were analysed using software Statistical Package for Social Sciences (SPSS) version 17.0, Inc. Chicago, IL, USA for the calculation of frequencies and percentages for categorical variables. The bivariate analysis involved the use of Chi-Square for testing the significance of associations between categorical variables. Furthermore, univariate logistic regression analysis was performed at the 95% confidence limit. The level of significance was set at P < 0.05.

## Results

### Sociodemographic characteristics of the population

Out of the 7,972 households selected for the study, 7,593 (95%) were successfully surveyed. The sampling was stratified by region and 48% of the surveyed households came from 4 regions: the Center (17%), the Far North (11%), Douala (10%), and the Northern (10%) regions. Since epidemiological facies are regional groupings, 70% of the surveyed people lived in the equatorial facies (made up of the Center, East, Littoral, North-West, West, South and South-West regions), 19% in the Sudano-Sahel facies (made up of the Adamaoua and North regions), and 11% in the Sahelian facies (made up of the Far North region) (Table 1).

**Table 1:** socio-demographic characteristics of the households surveyed

Variables	Category	Number of persons per category	Frequency per category (%)
Region	Adamaoua	487	8
	Center	990	17
	Douala	587	10
	East	396	7
	Far-north	678	11
	Littoral	368	6
	North	610	10
	Northwest	475	8
	West	518	9
	South	397	7
	Southwest	409	7
Facies	Sahelian facies	678	11
	Soudano-Sahelian facies	1097	19
	Equatorial facies	4140	70
Place of residence	Rural	3079	52
	Urban	2836	48
Sex	Female	1535	26
	Male	4380	74
Level of education	Without Level	1646	28
	Primary	1657	28
	Secondary 1st cycle	1196	20
	Secondary 2nd cycle	885	15
	University	496	8
Quintile of economic well-being	Very poor	901	15
	Average	1366	23
	Second	1241	21
	Fourth	1376	23
	Very rich	1031	17

Out of the respondents surveyed, 4,380 (74%) were male, 3,079 (52%) lived in rural areas, and 4,234 (78%) had at least elementary education. Regarding the standard of living, 901 (15%) were very poor and 1,031 (17%) were very rich (Table 1).

### Possession, usage and preferences of LLINs

Regarding the usage of LLINs, the inhabitants of 4743 (82%) households with at least one LLIN slept under the LLIN during the night before the survey (Table 2). The number of LLINs in a household is sufficient if universal coverage is reached, that is if there is 1 LLIN for every 2 people in the household. Following this criterion, 3772 (64%) households with at least 1 LLIN had sufficient LLINs. Respondents’ preference for one type of LLIN was assessed according to 4 criteria: the shape, colour, size, and texture. According to these criteria, 3,474 (59%) respondents had preferences for one type of LLIN. Specifically, 3261 (55%) had preferences for the shape of the LLIN, 3288 (56%) for the texture of the LLIN, 3305 (56%) for the colour, and 3345 (57%) for the size of the LLIN. The percentage of respondents that had preferences for all 4 types (shape, colour, size, and texture) was 50% (3020/5961) amongst the households surveyed.

**Table 2:** Possession and use of LLINs and household preferences surveyed

Variables	Category	Number per category	Fréquency (%)
Slept under a mosquito net last night (LLIN usage)	No	1075	18
	Yes	4743	82
number of LLINs sufficient in the household	No	2143	36
	Yes	3772	64
General preference for a certain type of mosquito net	No	2441	41
	Yes	3474	59
Preference for the shape of the mosquito net	No preference	2654	45
	Rectangular	2856	48
	Conical shape	405	7
Preference for the type of texture of the mosquito net	No preference	2627	44
	Rigid texture/polyethylene	401	7
	Soft texture/polyester	2887	49
Preference for the colour of the mosquito net	No Preference	2610	44
	Blue	1869	32
	White	891	15
	Green	367	6
	Pink	178	3
Preference for the size of the mosquito net	No preference	2570	43
	Long mosquito net (180 cm)	3239	55
	Short mosquito net (150 cm)	106	2

### Multivariate analysis of factors of LLIN usage

The multivariate analysis was done in 2 steps: the independence tests, and the logistic regression.

At the 5% threshold, there was a significant relationship between the use of LLINs variables and factors such as regions, facies, economic well-being, education level, sufficient LLINs, a general preference for a LLIN type, and preference for the shape and colour. The variables which indicated the place of residence, gender, fabric preference and size preference of LLINs did not have a statistically significant influence on the use of LLINs (Table 3).

**Table 3:** independence tests between variables and use of LLINs

Variables	Number of categories	Chi-square	P-value	Odds Ratio [CI 95%]
Regions	11	383.775	< 0.0001	
Facies	3	342.954	< 0.0001	
Level of well-being economic	5	73.66	< 0.0001	
Level of education	2	39.563	< 0.0001	0.639 [0.555–0.735]
Place of residence	2	0.959	0.916	1.069 [0.936–1.220]
Sex	2	1.076	0.8981	0.922 [0.792–1.074]
Sufficient number of LLINs	2	133.019	< 0.0001	2.179 [1.905–2.491]
General preference	2	4.06	0.0439	0.870 [0.760–0.996]
Preference of shape	2	4.612	0.0317	1.158 [1.013–1.324]
Fabric Preference	2	2.889	0.0892	1.123 [0.982–1.284]
Colour Preference	2	4.997	0.0254	1.165 [1.019–1.333]
size preference	2	3.598	0.0578	1.139 [0.996–1.303]

**Fig. 1:**
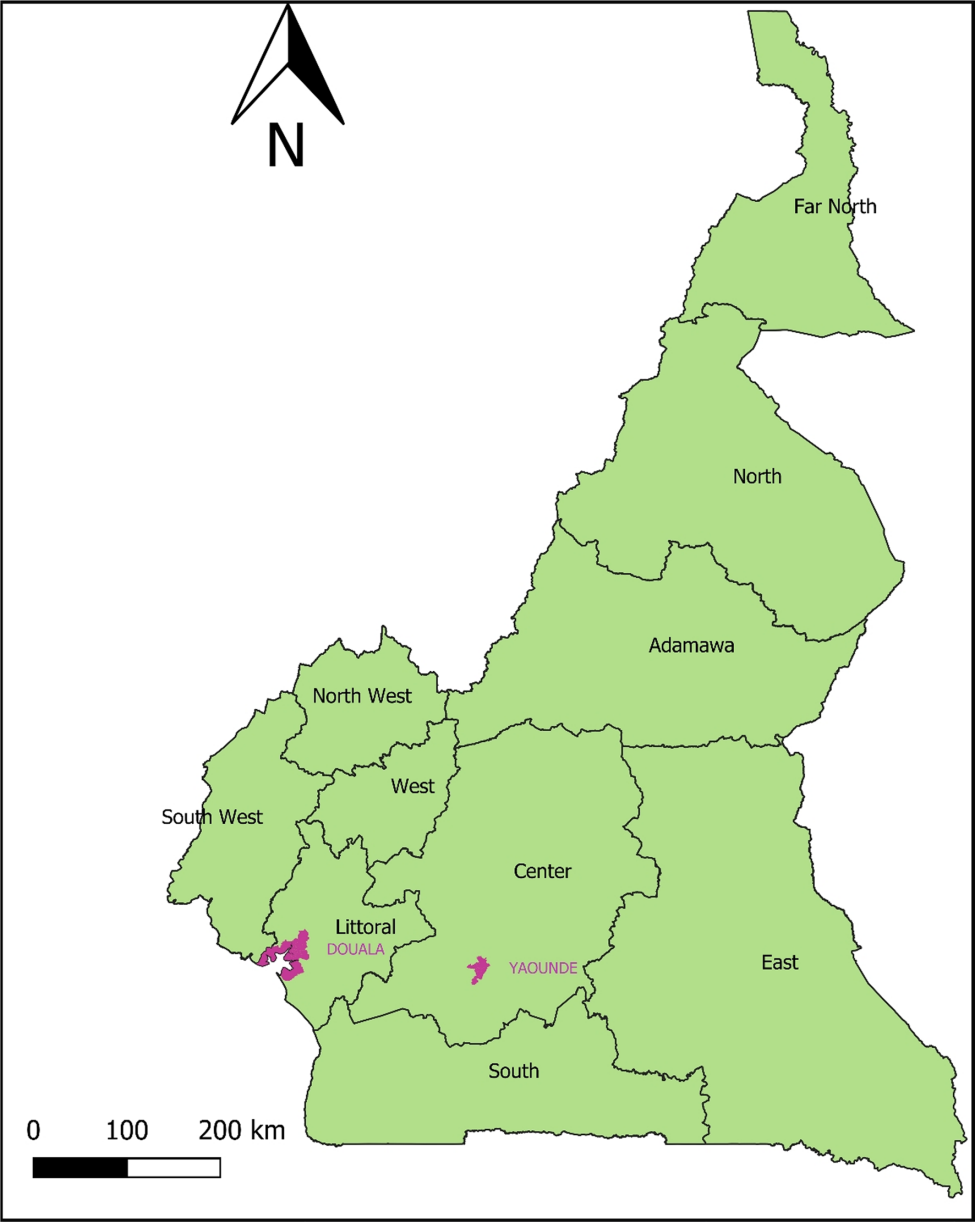
Map of Cameroon showing the ten regions of the country with the main cities (Yaounde and Douala)

The previous bivariate analysis did not account for the multi-collinearity between the variables and analyses the links 2 by 2 between the variables. To account for all the variables likely to have an influence on the use of LLINs, a subsequent analysis using a logistic model was made. The results are summarized in Table 4. This multivariate analysis with a logistic model excluded the variables gender, age, and size preference of LLINs since they were found above as not influencing the use of LLINs (see Fig. 1).

**Table 4:** Multivariate analysis with a logistic model combining all variables

Source	DDL	Khi^2^ (Wald)	Pr > Wald	Khi^2^ (LR)	Pr > LR
Region	10	219.60	< 0.0001	232.01	< 0.0001
Place of residence	1	0.92	0.3386	1.87	0.1716
Sufficient number of LLINs	1	112.32	< 0.0001	113.06	< 0.0001
Education level	4	23.25	0.0001	23.92	< 0.0001
Preference of LLIN shape	2	1.90	0.3874	2.83	0.2432
Preference of LLIN fabric	2	23.17	< 0.0001	23.38	< 0.0001
Preference of LLIN colour	4	4.20	0.3791	5.15	0.2724
Quintile of economic well being	4	10.83	0.0286	11.71	0.0197

By using the criteria of Wald and Lagrange at the 5% threshold, it appeared that the variables which influenced the use of LLINs were the region, the presence of a sufficient number of LLINs in households, the level of education of the head of household, the LLIN fabric, and the standard of living of the households. The variables indicating the place of residence and the shape and colour preference of LLINs did not have a statistically significant influence on the use of LLINs (Table 4).

**Fig. 2:**
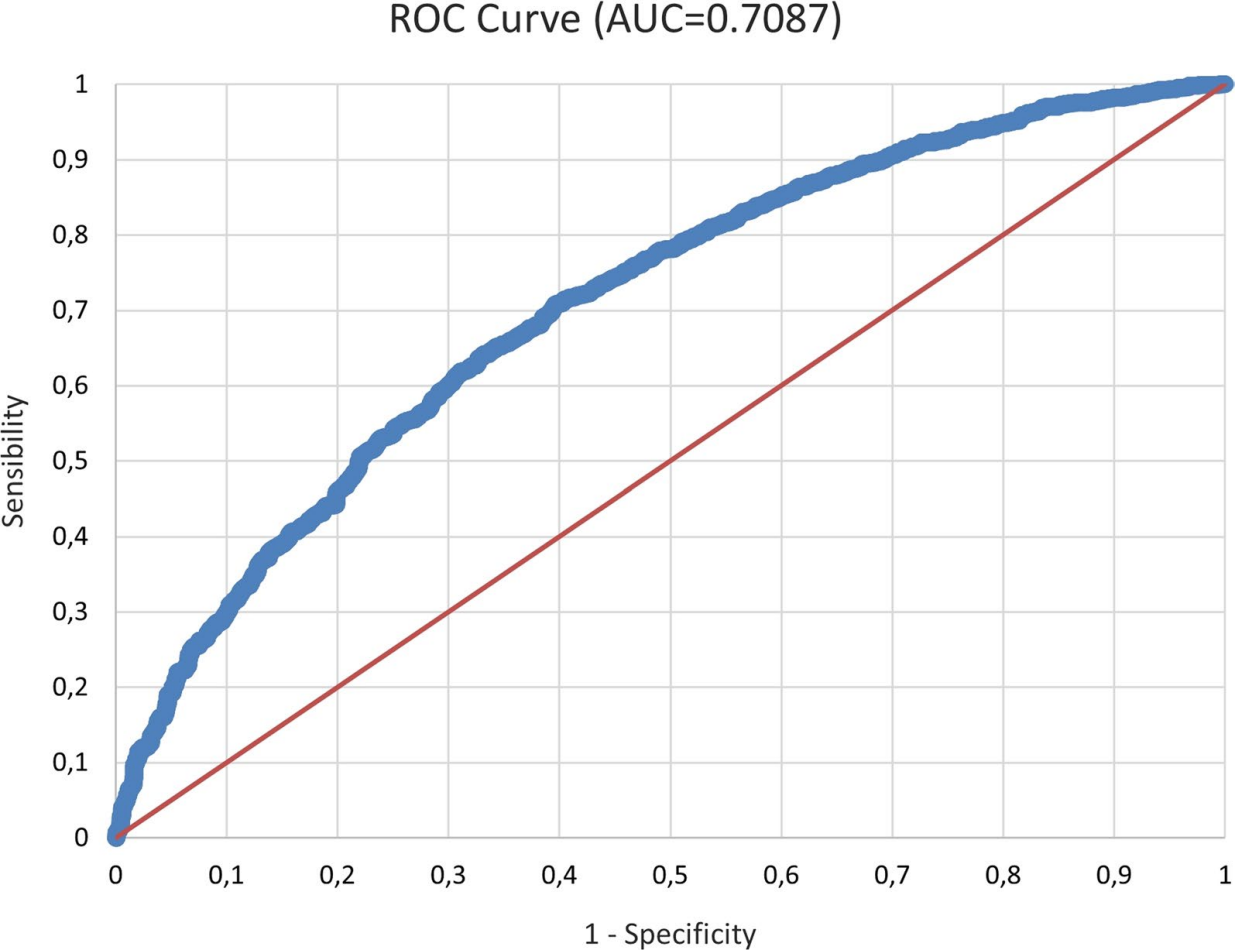
ROC Curve of the Variable A on the LLIN use, *Slept under the mosquito net last night*

To assess the quality of the model, the following statistics were used: the confusion matrix, the Area Under the ROC curve (AUC), and the Hosmer–Lemeshow test. From the confusion matrix, it emerged that the error rate was 18.01%, the sensitivity was 97.56%, the specificity was 13.70%, and the accuracy was 83.22%. The AUC was 0.7087, meaning that the capacity of discrimination of the model was acceptable. The Hosmer–Lemeshow test result was 12.22, corresponding to a p-value of 0.142 which is greater than the 5% threshold; this indicated that the model was verified and consistent with the data (Fig. 2).

Regarding the level of education, households whose heads of households were without any formal education had less LLINs than those who had primary level (OR = 1.38; 95% CI 1.12–1.78; p < 0.0028), secondary 1^st^ cycle (OR = 1.50; 95% CI 1.18–1.91; p < 0.0009), and secondary 2nd cycle education level (OR = 1.46; 95% CI 1.11–1.91; p < 0.0061). On the other hand, there was no statistically significant difference in LLIN use between households whose heads of households were without level and those with higher education level (OR = 0.92; CI 95% 0.67–1.27; p < 0.6145) (Table 5).

**Table 5:** Multivariate analysis with a logistic model per category of various variable

Source	Valeur	Erreur standard	Khi^2^ de Wald	*Pr > Khi^2^	*Odds ratio [IC 95%]
Constant	2.6839	0.2059	169.8888	< 0.0001	
region without-Adamaoua	0.0000	0.0000			
Center	− 0.5877	0.2119	7.6935	0.0055	0.5556 [0.3668–0.8416]
Douala	− 0.8209	0.2257	13.2255	0.0003	0.4401 [0.2827–0.6849]
East	− 0.9691	0.2284	18.0069	< 0.0001	0.3794 [0.2425–0.5936]
Far-north	− 2.0893	0.1938	116.1969	< 0.0001	0.1238 [0.0847–0.1810]
Littoral	− 0.8386	0.2384	12.3705	0.0004	0.4323 [0.2709–0.6898]
North	− 0.8129	0.2088	15.1527	< 0.0001	0.4436 [0.2946–0.6679]
North-west	− 1.1451	0.2203	27.0095	< 0.0001	0.3182 [0.2066–0.4901]
west	− 0.7136	0.2313	9.5147	0.0020	0.4899 [0.3113–0.7709]
South	− 1.5582	0.2251	47.8955	< 0.0001	0.2105 [0.1354–0.3273]
South-west	− 0.5850	0.2409	5.8959	0.0152	0.5571 [0.3474–0.8933]
Residence place-Urban	0.0000	0.0000			
Residence place-Rural	0.1053	0.1101	0.9157	0.3386	1.1111 [0.8955–1.3785]
adequate Number of LLIN -Yes	0.0000	0.0000			
adequate Number of LLIN –NO	− 0.7712	0.0728	112.3218	< 0.0001	0.4625 [0.4010–0.5333]
Education level- Without Level	0.0000	0.0000			
Primary	0.3235	0.1082	8.9294	0.0028	1.3819 [1.1177–1.7085]
Secondary 1st cycle	0.4075	0.1231	10.9642	0.0009	1.5031 [1.1809–1.9132]
Secondary 2nd cycle	0.3795	0.1384	7.5227	0.0061	1.4616 [1.1144–1.9169]
University	− 0.0824	0.1637	0.2537	0.6145	0.9209 [0.6681–1.2692]
Preference shape-None	0.0000	0.0000			
Rectangular	− 0.1563	0.2228	0.4920	0.4830	0.8553 [0.5527–1.3236]
Conic	− 0.3139	0.2519	1.5522	0.2128	0.7306 [0.4459–1.1971]
Preference Texture-None	0.0000	0.0000			
Soft texture /polyester	0.3829	0.2206	3.0137	0.0826	1.4665 [0.9518–2.2597]
Rough texture/polyethylene	− 0.2210	0.2435	0.8237	0.3641	0.8017 [0.4974–1.2921]
Colour preference-None	0.0000	0.0000			
Pink	− 0.4577	0.2673	2.9318	0.0868	0.6328 [0.3747–1.0684]
Blue	− 0.2605	0.1875	1.9304	0.1647	0.7706 [0.5336–1.1129]
White	− 0.2478	0.2005	1.5279	0.2164	0.7805 [0.5269–1.1562]
Green	− 0.1010	0.2311	0.1911	0.6620	0.9039 [0.5746–1.4219]
Quintile of economic well-being Second	0.0000	0.0000			
Very poor	− 0.0840	0.1253	0.4491	0.5028	0.9194 [0.7192–1.1755]
Average	0.1023	0.1253	0.6662	0.4144	1.1077 [0.8665–1.4161]
Fourth	− 0.0546	0.1503	0.1320	0.7164	0.9469 [0.7053–1.2712]
Very rich	− 0.3512	0.1731	4.1147	0.0425	0.7039 [0.5013–0.9882]

^*^The variables influencing the use of LLINs are those the p-value is less than 0.05 and the Odd ratio does not contain

Initially, four LLINs characteristics that could influence the use in households were identified: the shape (which could be rectangular or conical), the colour (which could be green, blue, white, or pink), the fabric (which could be flexible polyester or rough polyethylene), and the size of the LLIN (which could be long with 1.90 m in height or short with 1.5 m in height). After analysis, only the LLIN fabric had a statistically significant influence on their use in households. Households which preferred LLINs with a soft polyester texture were more likely to use LLINs than those who had no preference for texture (OR = 1.47; 95% CI 0.95- 2.26; p < 0.082). On the other hand, there was no statistically significant difference in use between households that had no texture preference and those which preferred LLINs with rough polyethylene textures (OR = 0.8; 95% CI % 0.50–1.30; p < 0.3641).

The availability of LLIN's also influenced the use of LLINs. Households that did not have sufficient LLINs had 54% less chance of using LLINs than those which had LLINs in sufficient quantities (OR = 0.46; 95% CI 0.40–0.53; P < 0.0001).

## Discussion

This study investigated the factors associated with net non-use of LLINs among households across the 10 regions of Cameroon following the completion of a mass LLIN distribution campaign in 2015–2016. Findings indicate that LLINs in sufficient quantity, the education level of the head of the household, the standard of living of the household, the texture preference of the LLIN fibre significantly influenced the LLIN use in the study population.

The use of ITNs produces enormous population-level benefits because they slow down malaria transmission by increasing mosquito death rates through the delay of feeding and/or the diversion of bites onto non-human hosts [46]. Even untreated nets provide some protection, while LLINs are approximately twice as effective as untreated nets [15, 47].

The current study shows the influence of the availability of a sufficient quantity of LLINs on their use. Indeed, living in a household with a sufficient number of LLIN to cover all members was a strong determinant of LLIN use. To achieve the availability of LLINs in sufficient numbers, the WHO recommends universal coverage, meaning that for each household 1 LLIN should be given for every two inhabitants [48]. This is corroborated by findings of a cross-national analysis of 15 survey datasets which showed that having sufficient intra-household access to an LLIN (defined as having ≥ 1 ITN per 2 household occupants) was a strong determinant of individual use of ITNs [49]. Our results show that in Cameroon, after the 2015–2016 universal mass distribution campaign, less than half (41,9%) of the households surveyed had at least one LLIN for every two inhabitants. This low universal coverage could be explained by the absence of household members during the census or the distribution phase which thus decreased the number of LLIN that were dispensed to each household [24]. The low universal coverage in Cameroon could also be because during the planning of the distribution campaign the number of LLIN allocated per household was limited, particularly for households of more than four members. Indeed, many post-campaign distributions surveys have reported that in instances where the maximum number of LLIN that one household could receive was limited, households with more than five residents were less likely to have sufficient LLIN to cover all occupants [50, 51].

The multivariate analysis with a logistic model revealed that the educational level of the head of household influenced the use of LLINs: the higher was the educational level, the greater was the use of LLINs in the household. This result was also observed in many studies across sub-Saharan African countries [40, 52, 53]. These studies describe that the level of education of the head of the household, in particular when it is a mother, influenced the use of LLIN by children under 5 years of age (U5): the low education level of mothers favoured LLIN non-use [54]. In contrast, García-Basteiro et al.found that the education level of the head of the household was not independently associated with ITN use by U5s [55]. Nevertheless, the results of intervention studies do demonstrate the positive effects of education on the use of LLINs. Indeed, a study on the school-based malaria education intervention engaging school children as health messengers revealed that LLIN use was greater among participants who had received malaria education compared to participants who did not receive any education [56].

Although many efforts have been made to improve LLIN access inequity between rich and poor, some disparities remained between socio-economic groups [57–61]. This study identified the standard of living of the household as one of the factors that influence the usage of LLIN: better access to LLIN by households seemed to increase with wealth, and this corroborates finding suggesting higher LLIN use among wealthier populations compared to poorer ones [59, 62]. However, this contrasts with the finding of large household surveys that have reported higher LLIN use among those living in households in the poorest wealth quintiles [63–65]. Nevertheless, households of wealthier standards might tend to use LLINs more often because they have better access to health information, and they can also afford to buy additional LLINs if need be.

Textile preferences were identified as a factor which could contribute to LLIN usage amongst the households. Head households preferred LLIN with softer textile (polyester) over ‘hard texture’ polyethylene nets, as also observed by Koenker et al. [66]. Moreover, by using a human-centred design approach to determine consumer preferences for LLINs Nets in Ghana, *Kim *et al. found that the material texture of polyethylene LLINs was perceived as both rough and hot while using polyester material for LLINs as it was perceived to be softer and less hot [67]. Unfortunately, some funders of malarial control programs do not give particular consideration to the users’ preferences concerning textiles or other characteristics like colour and shape; they argue that these do not impede rates of LLIN use in countries [66]. This study therefore provides tangible evidence for an argument in favour of a strong consideration for users’ preferences during the conception, fabrication, and purchase of the LLINs destined to mass distribution.

This study had certain limitations. It was a cross-sectional aetiological study and therefore it could not establish the causal link between the risk factors and the non-use of ITNs since it could not capture temporality. However, it would have been unethical to purposefully expose individuals to detrimental risk factors during a prospective study; hence, a cross-sectional study appeared as the adequate design to answer the research question of interest. Another limitation was that the sampling process was non-probabilistic, thus the study sample might not be representative of all the general population and could limit the generalizability of the results. Of note, like most developing countries, Cameroon does not possess granular data on its population given the absence of urban planning and systematic data recording on populations. Therefore, it was not possible to apply sampling weights during the sampling, hence the choice of a non-probabilistic sampling process. Furthermore, the surveyed households were selected across all the 12 regions of the country, and the resulting massive study sample size significantly decreased the probability of type II error.

This study had several strengths. This research was the first comprehensive study that uses rigorous statistical methods to estimate the prevalence and identify the risk factors of the non-use of ITNs which covers all the regions of Cameroon. Hence, these study results yield the first nationwide prevalence of the non-use of ITNs in Cameroon. Furthermore, the risk factors for the non-use of ITNs that were identified can inform health authorities and provide the foundations of nationwide policies aiming to improve the use of ITNs. Finally, this study provides a template for future evaluations of the use of ITNs in Cameroon as well as other sub-Saharan African nations with the same characteristics.

## Conclusions

The use of LLINs in Cameroon is low and varies according to some specific factors: the availability of LLINs in sufficient quantity, the education level of the head of the household, the standard of living of the household, and the texture preference of the LLIN fibre. These study results show that sufficient availability of LLIN is an exposure factor to its use, hence the need for universal coverage of the population in LLINs. To achieve and maintain that goal, regular mass campaign distributions should be organized. Furthermore, apart from antenatal distribution as means of continuous distribution of LLINs, other strategies like distribution to pupils and distribution to children during vaccination campaigns should also be taken into consideration. Moreover, populations’ preferences must be accounted for when ordering LLINs. Finally, communication must be intensified to sensitize households on the importance of regular LLIN use. A combination of these different actions will increase the use of LLINs and thus contribute to reduce the morbidity due to malaria transmission in Cameroon.

## Supplementary Information

**Additional file 1. **The database generated and used in the current study showing demographic characteristics information (including sex, age, date of birth, educational level) of household members, ownership and usage of nets, preferences and continuous use of the mosquito nets.

## Data Availability

The datasets generated and/or analysed during the current study are secured at the National Malaria Control Programme. These data are available upon official request to the director of the National Malaria Control Programme.

## Abbreviations

ITN: Insecticide-treated net
LLIN: Long-lasting insecticidal net
EA: Enumeration area
RGPH: General Population and Housing Census
WHO: World Health Organization
